# Assessing the information‐content of messy data to reconstruct population recovery dynamics for the world's rarest primate

**DOI:** 10.1002/ece3.70089

**Published:** 2024-08-07

**Authors:** Samuel T. Turvey, Erika Y. X. Lau, Clare Duncan, Heidi Ma, Hui Liu

**Affiliations:** ^1^ Institute of Zoology Zoological Society of London London UK; ^2^ Centre for Ecology & Conservation, Biosciences, College of Life and Environmental Sciences University of Exeter Cornwall UK; ^3^ School of Tropical Agriculture and Forestry Hainan University Haikou China

**Keywords:** bottleneck, data uncertainty, Hainan gibbon, *Nomascus hainanus*, population viability analysis, small population

## Abstract

Understanding the dynamics of population recovery in threatened species requires robust longitudinal monitoring datasets. However, evidence‐based decision‐making is often impeded by variable data collection approaches, necessitating critical evaluation of restricted available baselines. The Hainan gibbon, the world's rarest primate, had possibly declined to only seven or eight individuals in 1978 at Bawangling National Nature Reserve but has experienced subsequent population growth. Past population estimates lack detailed reporting of survey effort, and multiple conflicting estimates are available, hindering assessment of gibbon recovery. We investigated all reported estimates of Bawangling gibbon population size from 1978 to 2022, to evaluate the biological signal of population trends and the extent to which noise associated with varying survey effort, reporting and estimation may mask or misrepresent any underlying signal. This longitudinal dataset demonstrates that the Bawangling population experienced a series of bottlenecks and recoveries, with three successive periods of growth interspersed by population crashes (1978–1989, 1989–2000 and 2000–2022). The rate of gibbon population recovery was progressively slower over time in each successive period of growth, and this potential decline in recovery rate following serial bottlenecks suggests that additional management strategies may be required alongside “nature‐based solutions” for this species. However, population viability analysis suggests the 1978 founder population is unlikely to have been as low as seven individuals, raising concerns for interpreting reported historical population counts and understanding the dynamics of the species' recovery. We caution against overinterpreting potential signals within “messy” conservation datasets, and we emphasise the crucial importance of standardised replicable survey methods and transparent reporting of data and effort in all future surveys of Hainan gibbons and other highly threatened species.

## Graphical abstract

Reappraisal of Hainan gibbon historical data shows slower recovery after successive bottlenecks, suggesting escalating demographic impacts. However, past population estimates are unlikely to all be accurate, raising concerns for understanding the species' recovery dynamics. We highlight the risk of overinterpreting potential signals within historical datasets and the importance of analysing such data using appropriate frameworks that accommodate inherent noise and bias.

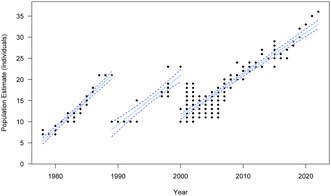

## 1 INTRODUCTION

Establishing robust baselines on population parameters is an essential component of evidence‐based conservation for threatened species (Salafsky et al., 2019; Sutherland et al., 2004). One of the most fundamental baselines needed to evaluate conservation effectiveness and guide management is an understanding of population dynamics through time, including changes in survivorship in response to different threats or interventions, and recovery rates following population declines. Large‐scale longitudinal monitoring datasets are typically needed to provide sufficient power to detect population change and predict future dynamics (Authier et al., 2020; Taylor et al., 2007; Taylor & Gerrodette, 1993; White, 2019). However, census datasets for many species are spatiotemporally incomplete and often contain survey data collected using variable uncoordinated methods and effort, hindering straightforward assessment of changes in population states and rates, and thus limiting the evidence available to identify suitable interventions and set realistic recovery targets (Kamp et al., 2016; Moussey et al., 2022; Scheele et al., 2019). Understanding population responses and dynamics through time can thus require critical evaluation and analysis of restricted available datasets using multiple approaches, to enable integration of both reconstructive hindcasting and predictive forecasting.

For species of extreme rarity, which are reduced to a handful of surviving individuals, determining past and future population dynamics is an urgent conservation priority needed to maximise the effectiveness of immediate responsive planning (Groombridge et al., 2004). Such populations have typically experienced recent demographic bottlenecks that result in loss of genetic diversity, increasing the risk of deleterious effects that compromise the ability to adapt to change and impede recovery (Briskie & Mackintosh, 2004; Frankham et al., 2010; Heber & Briskie, 2010). Reconstructing the magnitude, severity and frequency of past bottlenecks, and population‐level vulnerability or resilience to such events, is therefore crucial for understanding ongoing recovery dynamics and requirements for such species (Abascal et al., 2016; Jackson et al., 2022; Potter et al., 2020; Ramstad et al., 2013). However, populations at critically low sizes are often very hard to monitor or even detect, hindering the potential for systematic assessment of patterns and drivers of change over time (Black, 2020; Thompson, 2004). Incorporation of such uncertain data into decision support tools, such as demographic models used to infer population dynamics, can also be uninformative or misleading (Martin et al., 2022). Assessment of the quality, consistency, uncertainty and information‐content of available census data for species of extreme rarity is thus a crucial step that must underpin management inferences, predictions and recommendations about population response and recovery.

The Hainan gibbon (*Nomascus hainanus*) is the world's rarest primate and one of the rarest vertebrates (IUCN, 2022). Formerly distributed across Hainan Island, China (Liu et al., 1984; Turvey, Crees, & Di Fonzo, 2015), this species is now restricted to a single surviving population in Bawangling National Nature Reserve (BNNR; now a management area within Hainan Tropical Rainforest National Park). Other isolated gibbon populations across Hainan were extirpated by the late 20th or early 21st century (Fellowes et al., 2008; Liu et al., 1984; Turvey et al., 2017; Zhou et al., 2005). The population in the Bawangling region may have comprised c.150–200 individuals in the 1950s and early 1960s (Liu et al., 1984, 1987; Zhou et al., 2005). This population subsequently declined severely, primarily due to hunting, and “only seven or eight individuals were known to be alive” in 1978 (Liu et al., 1987). BNNR was established as a protected area in 1980 specifically to conserve the gibbons and their habitat (Chan et al., 2005), and the species has recovered over recent decades. It had reportedly reached 21 individuals by 1987–1989 (Liu et al., 1989) and comprised 36 known individuals in 2022 (Yang & Hu, 2022). However, the surviving Bawangling population shows greatly reduced genetic diversity and evidence of a recent bottleneck, with individuals related at the level of half‐ to full siblings between social groups (Bryant et al., 2016; Guo et al., 2020).

Although novel technologies (e.g., drones and passive acoustic detectors) are now being assessed for use in Hainan gibbon monitoring (Dufuorq et al., 2021; Zhang et al., 2020, 2023), all gibbon census surveying and past monitoring at Bawangling has been conducted using fixed‐point count methods (Brockelman & Srikosamatara, 1993), whereby survey teams listen opportunistically for gibbon calls at elevated listening posts and then locate and observe the animals (Bryant et al., 2017; Chan et al., 2005; Deng et al., 2017; Fellowes et al., 2008; Liu et al., 1989, 2022; Zhou et al., 2005). Population estimates based on direct observation are available for most years following the 1978 field survey and on an annual basis from 2000 onwards. No estimates have attempted to account for detection probability of gibbons or associated uncertainty of census results, and most studies are limited by a general lack of detailed reporting of survey effort or coverage (Bryant et al., 2017; Chan & Lo, 2023); it is therefore difficult to determine the relationship between numbers of detected individuals and total population size, hindering straightforward comparison of different estimates. Population estimates include both specific counts and ranges, and multiple different estimates provided by different surveys are available for many years, but in the absence of supporting information on survey methodology it is difficult to determine how precision of reported counts was determined and which estimate might be more accurate. However, attempts to reconstruct Hainan gibbon population trends have typically examined only temporal subsets of the available longitudinal data, and have selected a single specific census count per year rather than acknowledging or attempting to accommodate this data variation (Chan & Lo, 2023; Deng et al., 2017; Liu et al., 2022; Zhou et al., 2008; Zou et al., 2022). These assessments have inferred a continuous trajectory of population recovery over recent decades. Although concerns have been raised that recovery may not be at the maximum potential rate of increase (Zou et al., 2022), overall these studies are interpreted as providing strong support for current conservation management at BNNR and the adoption of “hands‐off” nature‐based solutions (Bleisch et al., 2020; Chan & Lo, 2023).

Understanding the full information‐content of available population data for the Hainan gibbon is of huge importance for maximally informed conservation planning, to assess whether population dynamics are more nuanced than previously assumed, whether predictive patterns about past recovery can provide lessons for the future, and whether the quality of available data is actually sufficient to enable conservation‐relevant inferences to be made about population responses. To address these concerns, we compiled a dataset of population estimates for the gibbon population at Bawangling from 1978 onwards, identified a series of population collapses and recoveries over recent decades, and analysed whether the rate of successive population recovery varied across different time periods. We also conducted population viability analysis (PVA) to determine whether the population could really have recovered from the initial bottleneck of only seven or eight reported individuals in 1978, and whether modelled recovery rates based upon known demographic parameters for the species match reported recovery rates to an initial peak of 21 individuals during the first time period of population growth between 1978 and 1987–1989. This comprehensive assessment allows us to address two key conservation questions: what conservation‐relevant biological signal is present in the Hainan gibbon population dataset, and to what extent does “noise” associated with varying survey effort, reporting and estimation mask or misrepresent any underlying signal?

## 2 METHODS

### 2.1 Population estimates and observed recovery rates

Our dataset included all available reported estimates of the size of the Hainan gibbon population at Bawangling across the 45‐year period from 1978 to 2022, derived from Chinese‐language and English‐language scientific papers, theses, grey literature reports and news articles (Table S1). We excluded estimates that were clearly erroneous on the basis of other available evidence, typically representing mistakes made when referring to data from previous studies (e.g., citing a different number to that given in a referred publication; citing publication year instead of survey year). Internal inconsistencies in census reporting by Liu et al. (1989) meant that multiple alternative estimates had to be calculated for 1982–1986 (Text S1).

We used linear regressions in R version 4.2.2 (R Core Team, 2022) to determine recovery rates for different discrete time periods that show gibbon population growth (Text S2). Regression slopes for different time periods were considered significantly different if their confidence intervals did not overlap. We used 83% confidence intervals for comparison, because using two sets of 95% confidence intervals provides an overly conservative test with extremely low probability of type I error (*α* < .01), whereas comparison of 83%–84% confidence intervals mimics statistical tests with *α* = .05 when confidence intervals do not overlap, for both symmetrical and asymmetrical confidence intervals (MacGregor‐Fors & Payton, 2013; Payton et al., 2003). We did not control for temporal autocorrelation in this analysis, as we are comparing change in the same population at the same site following repeated declines to a comparably low population size; the potential effect of existing temporal autocorrelation can thus be assumed to be constant across each time period.

### 2.2 Population modelling

We conducted PVA in Vortex version 10.5.5 (Lacy & Pollak, 2021), a modelling program designed specifically for mammalian and avian populations with low fecundity and long life spans. We used parameters previously used to model Hainan gibbon population viability in Bryant (2014) and Turvey, Traylor‐Holzer, et al. (2015) (Table S2). The demographic structure of the “seven or eight” gibbon individuals observed in 1978 was not reported by Liu et al. (1987), and Liu et al. (1989) later reported the sex and age class of only six of these individuals (two adult males born in 1970, two adult females born in 1970 and two juvenile females born in 1976). We therefore ran multiple models with alternative inferred demographic structures for the gibbon founder population, with demographically unknown individuals assigned to either sex and to one of the same two age classes (eight‐year‐old adult or two‐year‐old juvenile) reported by Liu et al. (1989). We ran three groups of model sets, with simulated founder populations of seven individuals (sets 1–4: addition of one demographically unknown individual), eight individuals (sets 5–14: addition of two individuals) and ten individuals (sets 15–24: addition of four individuals). For the first two model sets, we modelled all possible sex and age class combinations of extra individuals. For the ten‐individual model sets, we assigned the extra four individuals to all possible combinations of demographic categories in groups of two or four only, as this approach will capture the extremes of model likelihood. To further investigate the effect of founder population sex ratio on gibbon survival and recovery, we also ran an additional group of hypothetical counterfactual model sets for a population of seven individuals (sets 25–30), assuming the reported sex and age structure for six of the individuals was instead three adult males, and either two adult females and one juvenile female, or one adult female and two juvenile females; the single demographically unknown individual was again assigned to all combinations of either sex and to one of the same two age classes, to provide six novel scenarios (two of these eight combinations replicated previous scenarios). We ran all models using two different carrying capacities, *K* = 30 and *K* = 65, to model differing estimates of carrying capacity of available gibbon habitat at Bawangling in the 1980s and across the full 1978–2022 time period (Liu et al., 1989; Turvey, Traylor‐Holzer, et al., 2015; Wu et al., 2004). Sensitivity analysis of other Hainan gibbon demographic parameters was already conducted by Turvey, Traylor‐Holzer, et al. (2015), so was not repeated here.

For each of the 26 model sets, we ran four separate scenarios: for 9, 10 and 11 years, to match the range of time periods from 1978 to 1987–1989 reported by Liu et al. (1989) as the period over which the Bawangling gibbon population initially recovered to 21 individuals, and for 45 years, to match the full 1978–2022 time period that covers all of our population estimates. In total, we ran 120 scenarios (Table S3). Most scenarios were run twice, using different thresholds to define “extinction” in Vortex to allow us to investigate our two different questions: (1) only one sex remaining (all scenarios: to test likelihood of short‐term population survival following reported bottleneck size) and (2) critical population size <21 individuals (9–11 years scenarios only: to test likelihood of rate of population recovery from bottleneck to reported size by 1987–1989). All scenarios were run with 1000 iterations. No catastrophes were modelled, as we were interested in understanding the intrinsic rate of gibbon population increase and its relationship to observed data, rather than the additional effect of extrinsic events.

## 3 RESULTS

### 3.1 Population estimates and observed recovery rates

Our dataset contains at least one annual population estimate for the Bawangling gibbon population from 1978 to 2022, except for 1994, 1995, 1996 and 1999, when no data exist. Years where population data are available have a range of 1–10 different proposed census estimates (mean: 3.1), representing estimates reported by different surveys and uncertainty ranges reported by single surveys, with different reported values for a given year not necessarily representing consecutive number sequences. Only 11 years (24.4% of total time period from 1978 to 2022) have a single reported estimate.

**FIGURE 1:**
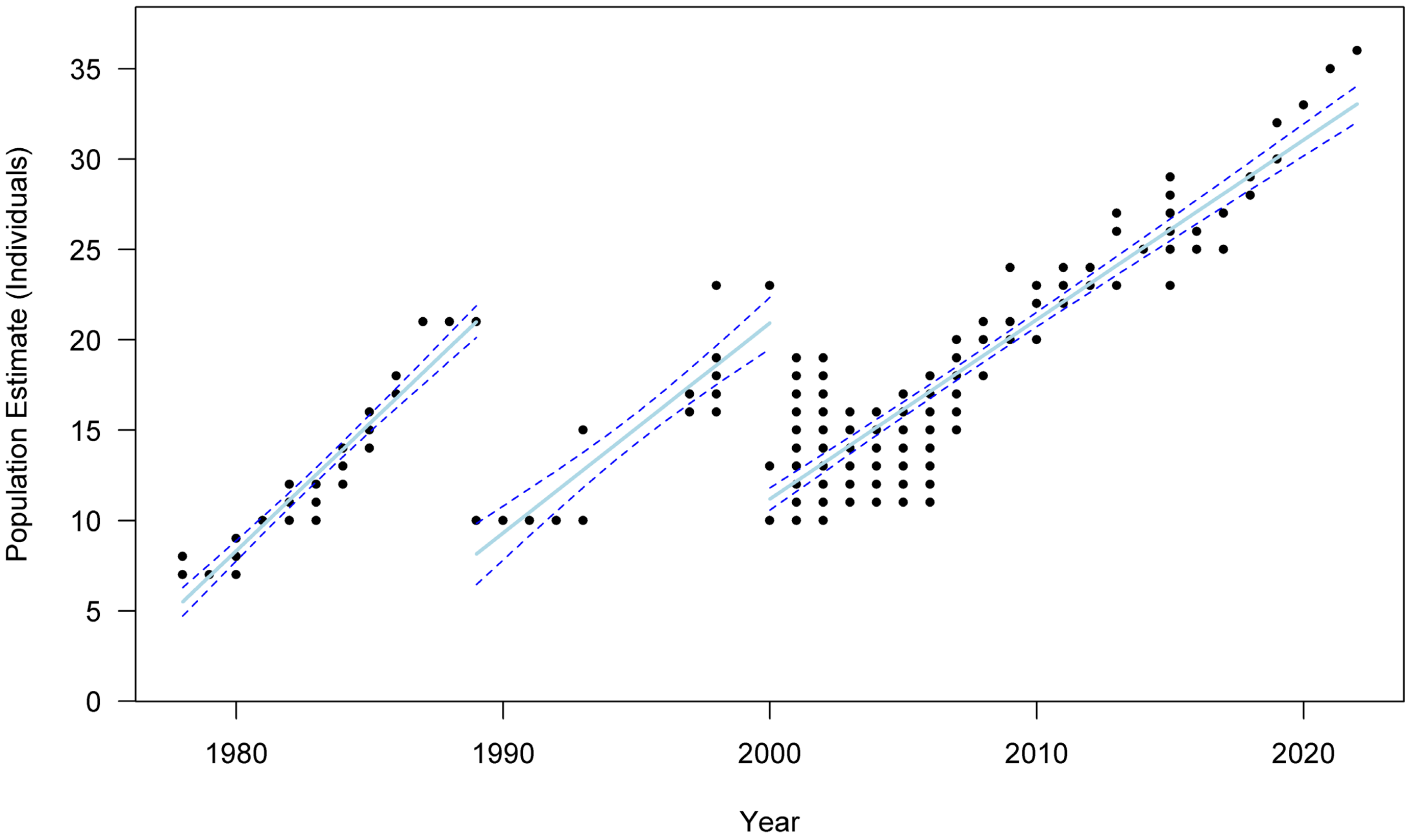
All reported annual population estimates for the Bawangling gibbon population from 1978 to 2022, showing three successive periods of recovery following population crashes. Regression slopes and 83% confidence intervals are fitted to each independent recovery period.

The dataset shows three successive periods of linear population growth in the Bawangling gibbon population (1978–1989, 1989–2000 and 2000–2022) (Figure 1). The first time period terminated with a population crash from an estimated 21 gibbons to 10 gibbons in 1989, and the second time period terminated with a crash from an estimated 23 gibbons to 10 or 13 gibbons in 2000. These two population collapses were documented by contemporary observers and are known to have been caused by poaching (Wu et al., 2004; Zhang, 1992; Zhang & Sheeran, 1994). In each case, population collapse is then followed by recovery.

**FIGURE 2:**
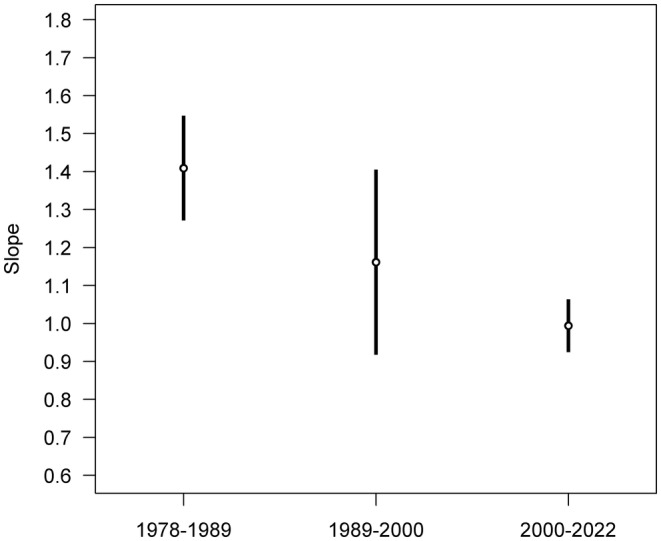
Slopes and 83% confidence intervals for three successive periods of gibbon population recovery at Bawangling between 1978 and 2022.

The slopes of each period of gibbon population growth decline successively from 1.409 in 1978–1989 to 1.161 in 1989–2000 and to 0.994 in 2000–2022 (Figure 2). The 83% confidence intervals of the linear regression slope for the second time period (1989–2000) are very wide, as this time period contains relatively few population estimates, and these confidence intervals overlap those for the first and third time periods. However, the 83% confidence intervals of the linear regression slopes for the first and third time periods are much narrower and non‐overlapping, and demonstrate that population recovery during 2000–2022 is significantly slower than during 1978–1989. These patterns thus show that overall rates of population recovery become shallower from 1978 to 2022.

### 3.2 Population modelling

Modelled extinction probability for the Bawangling gibbon population from its 1978 bottleneck to 1987–1989 was extremely low under either estimated carrying capacity, with probability of survival ≥0.977 for all combinations of inferred founder population size and 9–11 year scenarios (≥0.959 including counterfactual scenarios). The population showed similarly high survival probability (≥0.967) for all founder population size combinations and carrying capacities in the 45 year scenario (≥0.936 including counterfactual scenarios).

**FIGURE 3:**
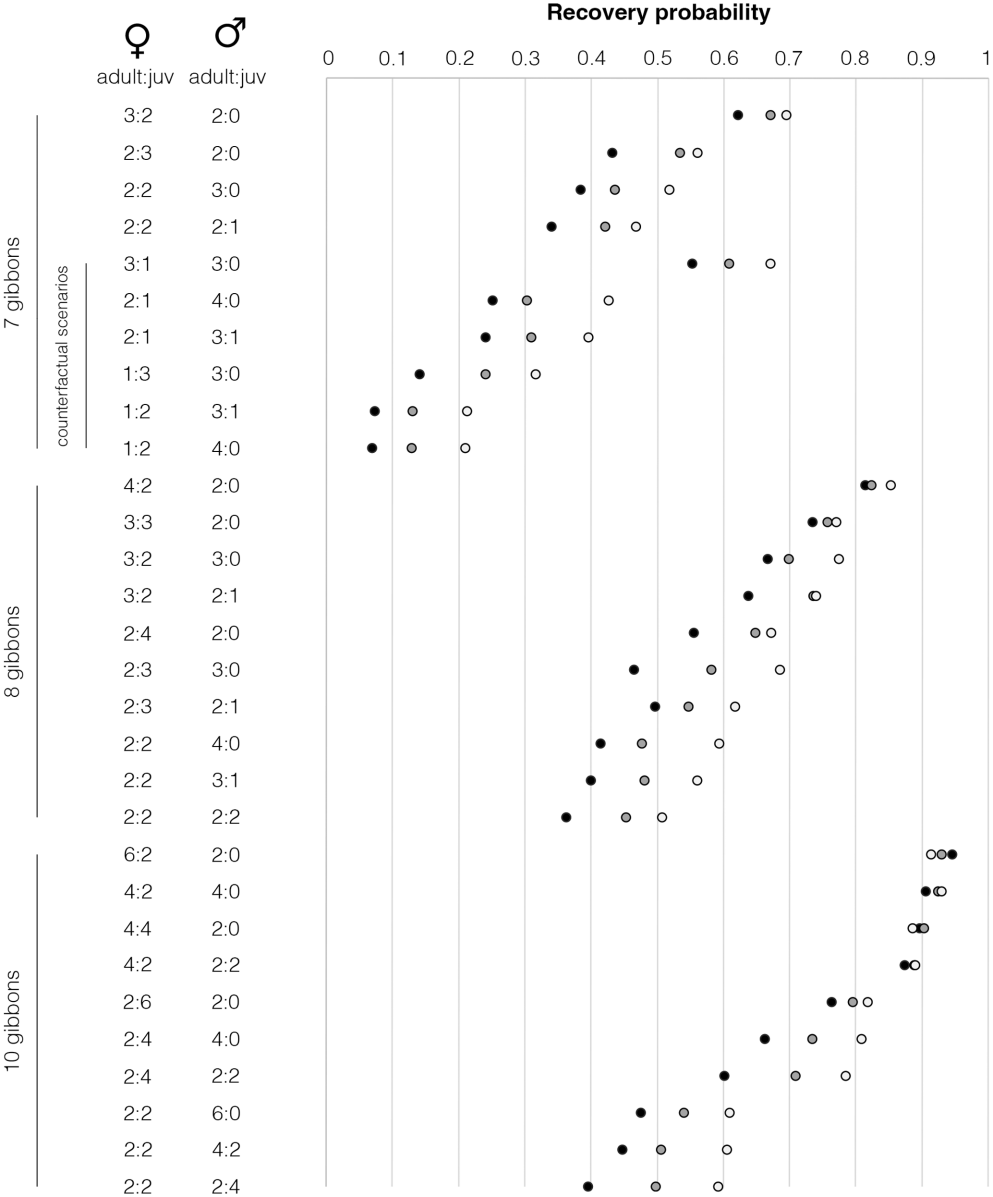
Simulated probabilities of Hainan gibbon population recovery from different founder population sizes in 1978 (seven, eight or ten individuals) to 21 individuals in 1987–1989, under different observed or counterfactual demographic structures. Key: dark grey, 9 year scenario; mid‐grey, 10 year scenario; pale grey, 11 year scenario.

However, the probability that founder populations with different starting sizes and demographic compositions could recover to 21 individuals by 1987–1989 was much more variable. Using observed demographic population structures and an estimated carrying capacity of 30 individuals in the 1980s, a founder population of only seven individuals had a probability of 0.340–0.621 of reaching the reported population size of 21 individuals by 1987, 0.421–0.670 by 1988 and 0.468–0.695 by 1989, whereas a population of eight individuals had a probability of 0.362–0.814 by 1987, 0.453–0.823 by 1988 and 0.507–0.853 by 1989, and a population of ten individuals had a range of probabilities of 0.396–0.945. Alternative recovery probability estimates using a higher carrying capacity of 65 individuals were very similar (Table S3). Across these scenarios, populations with more adult females consistently had the highest predicted recovery rates (Figure 3). This pattern is emphasised by the counterfactual models, which showed very low (<0.5) recovery probabilities across all hypothetical scenarios that included only two adult females (*K* = 30, 0.240–0.426; *K* = 65, 0.229–0.377) or one adult female (*K* = 30, 0.069–0.316; *K* = 65, 0.060–0.315) (Figure 3; Table S3).

## 4 DISCUSSION

Our study provides the first critical appraisal of the entirety of the data that are available to determine the past population trajectory of one of the world's rarest species, the Hainan gibbon, from the first census of the remnant Bawangling gibbon population in 1978 onwards. Our findings have important implications for understanding what has happened to the last Hainan gibbon population and how it might respond and recover in the future, with key implications for defining management priorities under different scenarios. More widely, they also provide guidelines for how to interpret evidence of varying quality on the demographic trajectory of other threatened species within data‐limited systems.

### 4.1 Conservation implications of the biological signal in the population dataset

Instead of exhibiting continuous growth following its first census in 1978 and subsequent establishment of protective measures, the demographic pattern shown by available survey data indicates that the Bawangling population experienced a series of successive bottlenecks and recoveries. The population crashes in 1989 and 2000 were caused by a resurgence of illegal logging and poaching within the reserve, possibly driven by black market demand for wildlife products; this occurred during the period of administrative reform when Hainan became an independent province, which caused a temporary lack of financial support for reserve management, patrolling or gibbon monitoring (Chan et al., 2005; Deng et al., 2017; Wang, 1995; Wu et al., 2004; Zhang, 1992; Zhang & Sheeran, 1994; Zhou et al., 2005). Fortuitously, population collapses were followed by recovery rather than extinction through demographic or environmental stochasticity, which may be particularly likely for a social group‐living species at risk from Allee effects (Feng et al., 2022).

It is possible that inherent demographic characteristics of the Bawangling population predisposed it to be somewhat resilient to extinction. The species has an interbirth interval of c.24 months, which is shorter than most other gibbons and thus enables more rapid recovery (Deng et al., 2017; Zhou et al., 2008). The Bawangling population has also exhibited a polygynous (1 male–2 females) mating system during recent decades (Wu et al., 2004; Zhou et al., 2008), which probably represents the species' natural population structure rather than an artefact of suboptimal habitat availability (Bryant et al., 2015), and the population has exhibited greater mating system flexibility as social groups have increased (Li et al., 2022). Polygynous and flexible mating systems are both associated with reduced stochastic extinction risk in some scenarios (Leach et al., 2020; Plesnar‐Bielak et al., 2012), although the relationship between mating system and extinction likelihood is complex, with polygynous systems sometimes having amplified risk (Lee et al., 2011; Lootvoet et al., 2015). Furthermore, four of the six individuals for which demographic data were recorded in 1978 were females (Liu et al., 1989). Female‐biased gibbon founder populations show greater likelihood of rapid recovery in our PVA modelling, and counterfactual simulations containing fewer adult females show much lower likelihood. However, whereas female‐biased founder population structure influences recovery rate, it has little effect on population survival over 45 years in our models, possibly because this time period constitutes few gibbon generations and thus represents a limited time for extinction from demographic factors. Indeed, the Bawangling population may have only bounced back repeatedly from perilously low numbers through sheer good luck (e.g., avoidance of deleterious stochastic processes such as typhoons, disease outbreaks or accidents; Bryant & Turvey, 2017), which may not always continue to prevail despite increased habitat protection and hunting bans. Its history of population survival thus provides no room for complacency in the future.

Furthermore, whilst the population has recovered from successive bottlenecks, survey data indicate a concerning longitudinal recovery trend. The three distinct recovery time periods across 1978–2022 show relatively similar population growth slopes (between 0.994 and 1.409), but these slopes become progressively lower over time. Although recovery during the most recent time period (2000–2022) has reached the highest modern gibbon population size at Bawangling, this reflects a longer period of continuous recovery thanks to improved protection, and this growth actually exhibits a statistically slower recovery rate compared to growth from 1978 to 1989. Identifying potential reasons for this worrying demographic pattern is thus of primary importance.

As our models show that recovery rate is strongly influenced by founder sex ratio, variation in recovery following successive bottlenecks might simply reflect chance variation in how many females survived each population crash. Unfortunately, this hypothesis is difficult to test, because the adult and juvenile sex ratio following past crashes was incompletely reported (Chan et al., 2005; Wang, 1995; Zhang & Sheeran, 1994; Zhou et al., 2005, 2008). Other potential hypotheses might also account for observed variation in recovery. Although BNNR encompasses almost 300 km^2^, its forest habitat is fragmented, with limited landscape connectivity presenting a challenge for long‐term gibbon population expansion (He et al., 2022; Zhang et al., 2010). Previous assessments have interpreted the Bawangling population as being restricted to a <15 km^2^ patch of primary forest around Mt Futouling (Chan et al., 2005; Turvey, Traylor‐Holzer, et al., 2015; Zhou et al., 2008). Hainan gibbon home range and habitat requirements remain incompletely understood (Bryant et al., 2017), but it is possible that recovery at Bawangling has slowed as the population approached the available habitat's carrying capacity (Turvey, Traylor‐Holzer, et al., 2015). This scenario highlights the urgency of increasing habitat connectivity to reduce the risk of density‐dependent limitations on recovery, through long‐term forest restoration and potentially also through temporary short‐term solutions such as canopy bridges (Chan & Lo, 2023; Chan, Lo, Hong, et al., 2020; Fellowes et al., 2008). However, in 2019 a new social group became established within secondary forest c.8 km north of Mt Futouling, demonstrating the potential for wider dispersal and broader habitat utilisation by the species across this landscape and suggesting that carrying capacity is higher than previously thought (Chan, Lo, & Mo, 2020). Some other recovering primate species have also increased their carrying capacity by expanding their habitat (Strier & Ives, 2012).

It is also possible that reduction in recovery rate across successive time periods might reflect an escalating demographic impact of the sequential genetic bottlenecks experienced by the Bawangling population since the 1970s. Tiny populations are likely to exhibit deleterious demographic consequences of reduced genetic diversity and inbreeding depression (Jamieson et al., 2006; Spielman et al., 2004), and single or serial bottlenecks are shown to result in declines in observed or predicted population growth across numerous threatened species (Beissinger et al., 2008; Grossen et al., 2018; Jackson et al., 2022; Leberg & Firmin, 2008; Weiser et al., 2016; White et al., 2015). The demographic mechanism by which inbreeding depression might impact the Bawangling population is difficult to determine, as detailed comparative data on population structure and breeding success are not consistently available from 1978 onwards. Individual females in all social groups have produced offspring at a consistent rate approximately every 2 years for the past two decades (Deng et al., 2017), although it is also suggested that the overall population is substantially below its full reproductive potential (Liu et al., 2022). There are also concerns about a possible male‐biased offspring sex ratio, although field observations are complicated by the fact that all juveniles resemble adult males in pelage coloration (Bryant et al., 2016; Deng et al., 2017; Fellowes et al., 2008; Liu et al., 1989). It is challenging to differentiate potential inbreeding effects in the Bawangling population from environmental effects of poor habitat quality and limited resource availability, which are also incompletely understood (Deng et al., 2017; Liu et al., 2022; Wang et al., 2022). Although very low heterozygosity has been demonstrated in the surviving population, and its possible impacts on long‐term viability and survival have been assessed through preliminary modelling (Turvey, Traylor‐Holzer, et al., 2015), the potential consequences and management implications of recent serial bottlenecks have not been considered fully in Hainan gibbon conservation planning. More rigorous assessment of genomic erosion in the Bawangling population and its conservation rescue implications represents an important research priority (cf. Jackson et al., 2022).

### 4.2 Does data noise obscure the biological signal of gibbon recovery?

It is essential to recognise the potential for misinterpretation of apparent demographic patterns in longitudinal data for the Bawangling gibbon population. To evaluate the true conservation information‐content of past survey data, it is necessary to critically consider whether these patterns might actually be artefacts of variable data quality. Are they merely “noise” rather than “signal”? Whereas previous assessments of Hainan gibbon population trends have assumed a single census estimate per year, most years have multiple different estimates, typically provided by different surveys and sometimes reflecting internal inconsistencies in data reporting; conversely, most surveys provide a single reported count rather than an uncertainty range (Figure 1; Table S1). It is effectively impossible to evaluate the likely relative accuracy of different competing estimates for any given year, due to a general lack of reporting of survey effort or methods (Bryant et al., 2017; Fellowes et al., 2008). Meaningful consideration of Hainan gibbon recovery trends must therefore address rather than ignore the challenge posed by data variability and lack of validation.

Overall, increased data noise is more likely to make underlying demographic patterns harder to discriminate (type II error) rather than generate spurious patterns (type I error), meaning that any patterns we can still detect are likely to be ecologically “real”. For example, the three time periods showing discrete periods of population recovery are associated with differing amounts of data; the second time period (1989–2000) has the lowest number of available population estimates, and its slope resultantly has wide confidence intervals that reduce statistical power to discriminate differences from the slopes of other time periods. However, other possible sources of variation between different estimates, such as the potential risk of double‐counting gibbons due to a lack of simultaneous wide‐range surveys and unexpressed assumptions about home range size (Fellowes et al., 2008), cannot be controlled for and could introduce unpredictable biases (cf. Dobson et al., 2020), raising inevitable uncertainty about any conclusions that can be made from existing data.

We also provide a note of caution about whether the 1978 founder population could really have been as low as seven individuals. Our results demonstrate that gibbons are extremely unlikely to have died out between 1978 and 1987–1989 in the absence of extrinsic stressors (e.g., hunting, storms, disease); this is unsurprising, as gibbons are long‐lived primates (Chivers et al., 2013), and the brief 9–11 year interval provides insufficient time for intrinsic demographic factors to impact the population. However, most scenarios with seven founders have a <0.5 probability of reaching the observed count of 21 individuals by 1987–1989, with much greater recovery probability from a larger founder size. It is therefore important to differentiate between the “seven or eight” gibbons documented by Liu et al. (1987) and the unknown actual size of this founder population. This consideration has significant wider implications for interpreting the accuracy of reported historical population counts in terms of unknowable past detection probabilities, and thus for properly understanding the dynamics of the species' recovery.

### 4.3 Conservation lessons from messy data

Our assessment of the information‐content of past Hainan gibbon population estimates provides a new baseline for understanding historical and potential future recovery trends in this Critically Endangered species, and highlights new directions for targeted conservation research and management planning. Importantly, whilst some tiny populations of highly threatened species have recovered without intensive conservation manipulations (Groombridge et al., 2009; Impey et al., 2002), the potential decline in Hainan gibbon recovery rate following serial bottlenecks raises suggestions that additional management strategies may need to be considered alongside “nature‐based solutions” for this species. Conversely, if the 1978 founder population was greater than seven or eight individuals, then the recovery slope for the first time period will become lower, reducing the likelihood of a true decline in successive recovery rates. This consideration demonstrates how failure to consider detectability of individuals within tiny remnant populations risks making erroneous conclusions about population dynamics and management implications. More widely, our study also emphasises the need for comparative assessment of recovery patterns for other bottlenecked species of extreme rarity, to understand commonalities and differences in population dynamics and trends in response to intrinsic and extrinsic factors.

“Messy data”, datasets that were not collected using a formal study design (e.g., unstructured or opportunistic collection of observations by ranger patrols or citizen science studies), are often the only source of information for conservation planning (Dobson et al., 2020). Our investigation of Hainan gibbon population data provides a case study for how to review existing datasets (cf. Murphy & Weiland, 2019), and how to address unmeasured but inherent noise and bias using appropriate quantitative frameworks that can accommodate or explore such data variation. We caution against overinterpreting potential signals within such datasets at face value, and we emphasise the crucial importance of using standardised replicable survey methods and of complete transparent reporting of survey data and effort in all future surveys of Hainan gibbons and other highly threatened species. If this approach is not followed, key metrics such as detection probabilities cannot be determined and such data cannot be used to guide conservation planning effectively. We also highlight the importance of being explicit about underlying assumptions, value judgements and fact claims that might be used to guide estimates of population status and recovery, which represent a further source of bias and error (Treves et al., 2021). Similar concerns have been raised for other remnant populations (Auriga Nusantara, 2023), and mischaracterisation, misinterpretation and misrepresentation of conservation scenarios through errors in engaging with available data are wider problems across environmental decision‐making and policy (Challender et al., 2022). To achieve these key goals, we encourage greater collaboration and data‐sharing for the Hainan gibbon and other threatened species, to maximise understanding of conservation‐relevant data and prevent time and resources from being wasted (Haddaway, 2015; Mace et al., 2000).

## AUTHOR CONTRIBUTIONS

**Samuel T. Turvey:** Conceptualization (lead); data curation (lead); formal analysis (supporting); funding acquisition (lead); investigation (lead); methodology (lead); project administration (lead); resources (lead); supervision (lead); validation (lead); visualization (lead); writing – original draft (lead); writing – review and editing (lead). **Erika Y. X. Lau:** Data curation (supporting); formal analysis (lead); investigation (supporting); methodology (supporting). **Clare Duncan:** Formal analysis (supporting); investigation (supporting); methodology (supporting); resources (supporting); validation (supporting); visualization (supporting); writing – review and editing (supporting). **Heidi Ma:** Data curation (supporting); funding acquisition (supporting); project administration (supporting); resources (supporting); supervision (supporting). **Hui Liu:** Data curation (supporting); project administration (supporting); resources (supporting); supervision (supporting).

## CONFLICT OF INTEREST STATEMENT

The authors have no competing interests.

## Supporting information


Text S1.

Text S2.

Table S1.

Table S2.

Table S3.

## Data Availability

All supporting data are available in the Supplementary Material.
