# Supplementary material for: Assessing the information‐content of messy data to reconstruct population recovery dynamics for the world's rarest primate
Source: Ecol Evol. 2024 Aug 7;14(8):e70089. doi: 10.1002/ece3.70089 (PMC11303811; doi:10.1002/ece3.70089)
Supplement: Supplementary file 1 — Text S1. [file ECE3-14-e70089-s002.docx]

**Text S1: Calculating gibbon population growth in the 1980s**

The only source of information about gibbon population size and growth at Bawangling for much of the 1980s is Liu et al. (1989). However, the population tallies provided in this study unfortunately do not match up with each other, necessitating estimation of a range of possible gibbon population sizes between 1982 and 1987.

Liu et al. (1989) state that “the total population has grown from 10 individuals in early 1984 to 21 in 1987” (p. 251). They also state that “Since 1982, 12 infants have been born … One birth occurred in 1982, two in 1984, two in 1985, three in 1986, and three in 1987” (p. 253). However, even just accounting for these 11 dated births, and assuming an accurately-reported population size of 10 individuals at the start of 1982 and 21 individuals by the end of 1987 (allowing for births within each year), these different population estimates do not add up with each other:

|  |  | ***10 in 1984*** | ***21 in 1987*** |
| --- | --- | --- | --- |
|  |  | ***Adding births*** | ***Subtracting births*** |
| 1982 | one birth | **10** | **10 --> 11** |
| 1983 | no births | **10** | **11** |
| 1984 | two births | **10 --> 12** | **11 --> 13** |
| 1985 | two births | **12 --> 14** | **13 --> 15** |
| 1986 | three births | **14 --> 17** | **15 --> 18** |
| 1987 | three births | **not 21!** | **18 --> 21** |

Accommodating the date of the unreported twelfth birth adds further confusion to population estimates. Liu et al. (1989) state that “Eleven of these 12 young were still alive in the spring of 1989” (p. 253), and “A juvenile disappeared at the end of 1986 for unknown reasons” (p. 251). So, if we assume that this extra individual was the animal not reported in the annual birth tally discussed above, it could presumably have been born within any of the year range where Liu et al. (1989) also reported other gibbon births occurring at Bawangling. This gives a range of possible estimates for the year in which this twelfth birth occurred, before the reported death of a juvenile in 1986:

|  |  | ***10 in 1984*** | ***21 in 1987*** |  |  |  |
| --- | --- | --- | --- | --- | --- | --- |
|  |  | ***Adding births*** | ***Subtracting births*** | |  |  |
| 1982 | one birth | **10** | **10 --> 11** | one birth + extra 1 | | **max 12** |
| 1983 | no births | **10** | **11** | no births + extra 1 | | **max 12** |
| 1984 | two births | **10 --> 12** | **11 --> 13** | two births + extra 1 | | **max 14** |
| 1985 | two births | **12 --> 14** | **13 --> 15** | two births + extra 1 | | **max 16** |
| 1986 | three births | **14 --> 17** | **15 --> 18** | three births, extra juv dies | | **18** |
| 1987 | three births | **not 21!** | **18 --> 21** | three births |  | **21** |

This estimation thus provides a range of possible counts for the total number of gibbons present at Bawangling between the end of 1982 and 1987, with no obvious means to infer which annual estimate is more likely to be correct:

|  | Minimum | Maximum |
| --- | --- | --- |
| 1982 | 10 | 12 |
| 1983 | 10 | 12 |
| 1984 | 12 | 14 |
| 1985 | 14 | 16 |
| 1986 | 17 | 18 |
| 1987 | 21 | 21 |

We therefore use all values within each possible count range for each year in our study’s population modelling analyses, as there is no *a priori* way to select or rank the likelihood of one annual estimate over another.

**Reference**

Liu, Z., Zhang, Y., Jiang, H., & Southwick, C. (1989). Population structure of *Hylobates concolor* in Bawanglin Nature Reserve, Hainan, China. *American Journal of Primatology*, *19*, 247–254.
