# Supplementary material for: Assessing the information‐content of messy data to reconstruct population recovery dynamics for the world's rarest primate
Source: Ecol Evol. 2024 Aug 7;14(8):e70089. doi: 10.1002/ece3.70089 (PMC11303811; doi:10.1002/ece3.70089)
Supplement: Supplementary file 2 — Text S2. [file ECE3-14-e70089-s005.docx]

**Text S2: R code used for linear regressions with 83% confidence intervals of gibbon population growth across discrete time-periods**

install.packages("stringr")

library("stringr")

basedir<-"F:/Current Work/GibbonPopulations/"

png(str_c(basedir,"GibbonPopulationTrends.png"), width = 22, height = 15, units = "cm", res = 600)

gg<-read.csv(str_c(basedir,"gibbon population dataset.csv"))

plot(gg$Population~gg$Year,pch=16,ylab="Population Estimate (Individuals)",xlab="Year",ylim=c(1.4,37),cex=0.75,las=1)

yearstart<-1978

yearend<-1989

dat<-subset(gg,(Year>=yearstart) & (Year<=yearend))

dat<-dat[-which(dat$Year=="1989" & dat$Population=="10"),]

yrs<-dat$Year-(min(dat$Year)-1)

mod<-lm(dat$Population~yrs,na.action=na.omit)

summary(mod)

lines(predict(mod)~dat$Year,lwd=2,col="lightblue")

conf_interval <- predict(mod, newdata=data.frame(yrs=yrs), interval="confidence",

level = 0.83)

lines(dat$Year, conf_interval[,2], col="blue", lty=2)

lines(dat$Year, conf_interval[,3], col="blue", lty=2)

slope1<-coef(mod)[2]

slope1

cis1<-confint(mod,level=0.83)[2,]

cis1

yearstart<-1989

yearend<-2000

dat<-subset(gg,(Year>=yearstart) & (Year<=yearend))

dat<-dat[-which(dat$Year=="1989" & dat$Population=="21"),]

dat<-dat[-which(dat$Year=="2000" & dat$Population=="10"),]

dat<-dat[-which(dat$Year=="2000" & dat$Population=="13"),]

yrs<-dat$Year-(min(dat$Year)-1)

mod<-lm(dat$Population~yrs,na.action=na.omit)

summary(mod)

lines(predict(mod,data.frame(yrs=yrs))~dat$Year,lwd=2,col="lightblue")

conf_interval <- predict(mod, newdata=data.frame(yrs=yrs), interval="confidence",

level = 0.83)

lines(dat$Year, conf_interval[,2], col="blue", lty=2)

lines(dat$Year, conf_interval[,3], col="blue", lty=2)

slope2<-coef(mod)[2]

slope2

cis2<-confint(mod,level=0.83)[2,]

cis2

yearstart<-2000

yearend<-2022

dat<-subset(gg,(Year>=yearstart) & (Year<=yearend))

dat<-dat[-which(dat$Year=="2000" & dat$Population=="23"),]

yrs<-dat$Year-(min(dat$Year)-1)

mod<-lm(dat$Population~yrs,na.action=na.omit)

summary(mod)

lines(predict(mod,data.frame(yrs=yrs))~dat$Year,lwd=2,col="lightblue")

conf_interval <- predict(mod, newdata=data.frame(yrs=yrs), interval="confidence",

level = 0.83)

lines(dat$Year, conf_interval[,2], col="blue", lty=2)

lines(dat$Year, conf_interval[,3], col="blue", lty=2)

slope3<-coef(mod)[2]

slope3

cis3<-confint(mod,level=0.83)[2,]

cis3

dev.off()

png(str_c(basedir,"GibbonPopulationTrends.png"), width = 15, height = 15, units = "cm", res = 600)

pad_plot = function(x, y, pad=0.175, n=5, ...) {

xrng = diff(range(x))

plot(x,y, xlim = range(x) + c(-1,1)*pad*xrng, xaxt="n", ...)

}

pad_plot(c(1,2,3),c(slope1,slope2,slope3),ylim=c(0.6,max(c(cis1,cis2,cis3)+0.25)),pch=16,xaxt="n",yaxt="n",ylab="Slope",xlab=" ")

lines(y=c(cis1),x=rep(1,2),lwd=3,lend=2)

lines(y=c(cis2),x=rep(2,2),lwd=3,lend=2)

lines(y=c(cis3),x=rep(3,2),lwd=3,lend=2)

points(c(1,2,3),c(slope1,slope2,slope3),ylim=c(0,max(c(cis1,cis2,cis3)+1)),pch=16,col="white",cex=0.5)

axis(side=1,at=c(1,2,3),labels=c("1978-1989","1989-2000","2000-2022"))

axis(side=2,at=round(seq(0.6,1.8,by=0.1),2),labels=c("0.6","0.7","0.8","0.9","1.0","1.1","1.2","1.3","1.4","1.5","1.6","1.7","1.8"),las=1)

dev.off()
